# Investigating Asthma Disparities in Hispanic Communities Using Machine Learning Algorithms on the *All of Us* Researcher Workbench

**DOI:** 10.3390/healthcare13233178

**Published:** 2025-12-04

**Authors:** Lei Jin, Rajesh Melaram

**Affiliations:** 1 College of Science, Texas A&M University—Corpus Christi, Corpus Christi, TX 78412, USA; 2 College of Nursing and Health Sciences, Texas A&M University—Corpus Christi, Corpus Christi, TX 78412, USA; rajesh.melaram@tamucc.edu

**Keywords:** access barriers, All of Us, asthma, BMI, disparities, Hispanic, machine learning, socioeconomic

## Abstract

**Purpose**: This study aims to examine factors associated with asthma prevalence among Hispanic participants in the United States, focusing on access barriers, socioeconomic indicators such as education and income, and BMI. Data from the *All of Us* Research Program were analyzed using both traditional statistical models and interpretable machine learning algorithms. **Methods**: We analyzed data from the *All of Us* Research Program, comparing individuals with and without asthma. Logistic regression models and interpretable machine learning algorithms, including MARS (Multivariate Adaptive Regression Splines) and CIT (Conditional Inference Trees), were used to identify factors associated with asthma prevalence and their interactions. **Results**: The logistic regression analysis identified several variables associated with higher odds of asthma, including older age, female sex, greater access barriers, higher BMI, lower income, and higher education levels. Hispanic participants with greater access barriers had 26.3% higher odds of asthma prevalence (aPOR = 1.263, 95% CI: 1.114–1.433) compared to those without such barriers, and each unit increase in BMI was associated with a 2.9% increase in the odds of having asthma (aPOR = 1.029, 95% CI: 1.023–1.035). The MARS algorithm captured nonlinear relationships and interactions, highlighting BMI, age, sex, access barriers, income, and education as key predictors associated with asthma prevalence. Among participants younger than 60.6 years, younger age was linked with higher asthma prevalence. An interaction between age (above 21.5) and male sex indicated that the odds of asthma slightly decreased with age among males. Additionally, low-income and high BMI together were associated with elevated asthma prevalence, suggesting compounding vulnerabilities. The CIT identified BMI as the most influential variable and further stratified asthma prevalence by age, sex, education, income, and access barriers. Higher asthma prevalence was consistently observed among older females with high BMI, lower income, and greater access barriers. **Conclusions**: Among Hispanic participants in the *All of Us* Research Program, lower income combined with higher BMI and greater access barriers were significantly associated with increased odds of asthma. Males had lower odds of asthma, while older individuals showed higher asthma prevalence. These findings highlight important associations rather than causal relationships and may inform public health efforts to address asthma disparities related to weight and healthcare accessibility among Hispanic populations.

## 1. Introduction

Asthma is a chronic inflammatory respiratory condition that requires ongoing medical management and contributes substantially to morbidity, mortality, and healthcare costs in the United States (U.S.). Consequently, asthma is also associated with lower quality of life, increased healthcare utilization, and decreased work productivity. Healthcare-related costs for asthma in the U.S. represent a societal burden, resulting in $50.3 billion in medical costs and $3 billion from missed school and workdays, based on yearly estimates between 2008 and 2013 [1].

According to recent findings [2], asthma prevalence among adults increased significantly between 2013 and 2021. As of 2022, approximately 26.8 million Americans, or about 8% of the population, have asthma [3]. The prevalence of asthma varies across racial and ethnic groups and is influenced by various factors such as physical characteristics, lifestyle behaviors, environmental pollutants, socioeconomic status, and geographic location [4,5].

Previous studies generally report lower asthma prevalence in rural areas compared to urban areas, particularly in young children and adults [6,7]. However, asthma prevalence among low-income minority residents in rural areas remains considerably high [8]. Rural minorities, including Hispanics, have lower levels of education (high school diploma/GED) and annual household incomes (<$25,000) compared to non-Hispanic Whites [9]. In a study consisting of 267 rural counties, 20% of poor rural Hispanics resided in counties with high and persistent poverty [10]. These findings reflect the role of socioeconomic indicators in asthma prevalence among Hispanics in rural areas.

Hispanics often face greater access barriers and social risk factors, which may contribute to their increased risk of asthma. Additionally, a higher prevalence of lack of insurance and cost-related unmet medical needs is persistently observed among Hispanics than among non-Hispanic Whites, based on evidence from 1999 to 2018 [11]. They are also more likely to encounter access barriers to medical care, including a lack of transportation, long waiting times, and inconvenient office hours [12]. Despite ongoing efforts in addressing these factors, it remains unclear how access barriers and their potential interactions with age, sex, BMI, and socioeconomic indicators such as education and income influence asthma prevalence in Hispanics.

The *All of Us* Research Program, a large-scale initiative led by the National Institutes of Health (NIH), provides a rich and diverse data resource. It integrates electronic health records, surveys, physical measurements, genomic data, and wearable device information. In the current *All of Us* Curated Data Repository (CDR) version 8, the dataset includes survey data from over 633,000 participants, physical measurements from more than 509,000, and electronic health records from over 393,000 individuals [13]. Researchers access the data through a secure, cloud-based Workbench that supports R and Python via Jupyter Notebooks in addition to SAS. For example, ref. [14] utilized this platform to examine racial differences in length of stay and hospital readmission for asthma. Another study investigated the impact of cost-related barriers on medication use faced by obesity-associated asthma via SAS [15].

The scale and diversity of the *All of Us* database make it especially well-suited for applying statistical and machine learning approaches to identify complex patterns and interactions in health outcomes such as asthma. Specifically, Multivariate Adaptive Regression Splines (MARS) and Conditional Inference Trees (CITs) can capture nonlinear relationships and interaction effects, while maintaining model interpretability. Compared with logistic regression, MARS and CITs offer greater flexibility by capturing nonlinear associations, detecting interactions, and identifying data-driven thresholds without strict parametric or linearity assumptions, providing a more complete yet still interpretable characterization of asthma-related patterns [16,17]. In addition, the Bonferroni adjustment in CITs helps control the overall Type I error rate, improving the reliability of the identified associations.

By integrating statistical and machine learning approaches with the computational infrastructure of the *All of Us* platform, this study addresses a key knowledge gap. Specifically, the analysis provides evidence on how access barriers interact with biophysical and sociodemographic characteristics to shape asthma prevalence among Hispanics. We hypothesize that higher asthma prevalence in Hispanic communities is influenced by greater access barriers, lower income and education levels, and higher BMI. These findings aim to support public health strategies that improve healthcare access and promote physical activity to reduce asthma-related disparities.

## 2. Methods

### 2.1. Data Overview

In this cross-sectional study, we investigated the prevalence of asthma in the Hispanic population and its associated factors using data from version 8 of the *All of Us* Research Program. All analyses were conducted within the secure *All of Us* Researcher Workbench platform, utilizing self-reported data from surveys, physical measurements, and electronic health records (EHRs). In the *All of Us* Research Program, asthma identification follows the Observational Medical Outcomes Partnership (OMOP) Common Data Model (CDM), which harmonizes clinical concepts across International Classification of Diseases (ICD-9/ICD-10), Systematized Nomenclature of Medicine—Clinical Terms (SNOMED CT), and other vocabularies. We defined asthma using the standardized OMOP concept for general asthma (OMOP concept ID 317009) together with all its SNOMED descendant concepts, which include ICD-9 (493) and ICD-10 (J45) mappings. Asthma status was determined from EHR condition records, and participants were classified as having asthma if their EHR included any diagnosis mapped to concept ID 317009 or its descendants. Individuals without any asthma-related medical concepts in their EHRs were considered non-asthmatic. The analysis included only Hispanic individuals with available EHR, and sociodemographic factors and access barriers derived from the Basic Survey and Healthcare Access and Utilization Survey, respectively.

Participants were classified as experiencing access barriers if they answered “yes” to either of the following questions in the Healthcare Access and Utilization Survey: (1) “Have you delayed getting care for any of the following reasons in the past 12 months? You live in a rural area where distance to the healthcare provider is too far,” or (2) “In the past 12 months, have any of the following been a problem for you in getting health care? Transportation to the doctor’s office.” Participants who did not participate in the survey were excluded from analyses. Note that experiencing access barriers here is related to a proxy measure, rurality. Although rurality is not directly defined in the *All of Us* dataset, one study used access barriers as a proxy for rural residence [18]. In our analysis, access barriers are included as the main independent variable.

Age was approximated using the participant’s age at the time of the survey, calculated as the difference between the date of the survey and the recorded date of birth, since the specific age at asthma diagnosis was not available for those without asthma.

Sociodemographic variables such as sex at birth (male or female), race, ethnicity, education level, and annual income were obtained from The Basics Survey, while insurance type was derived from the Healthcare Access and Utilization Survey. Body mass index (BMI) was obtained from physical measurement visits for each participant. For machine learning algorithms, ordinal variables such as income and education were converted to integer values to preserve their inherent order. Both income and education were originally collected as ordered categorical variables with multiple levels and were recoded as integers reflecting their underlying order (from lowest to highest) for use in the MARS and CIT analyses. In addition, to improve interpretability by modeling age in 10-year increments, we created the variable Age10, defined as Age divided by 10. Responses such as “Skip,” “Prefer not to answer,” or otherwise missing were coded as NA. These transformations and approximations were necessary due to the format of the original data and were implemented consistently to support valid and interpretable subgroup comparisons across the analysis.

### 2.2. Descriptive Analysis

To explore patterns in asthma prevalence and understand the distribution of key demographic and socioeconomic variables, we initially presented numerical summaries for Hispanic participants. Additionally, descriptive statistics summarized central tendencies and variability for continuous variables such as age and BMI. Collectively, these analyses provided an initial overview of the dataset, highlighting subgroups potentially with a higher prevalence of asthma.

### 2.3. Imputation

Different methods of missing value imputation including KNN imputation, median/mode imputation, replacing missing values with a separate ‘NA’ category, and complete-case analysis were performed for sensitivity analysis. KNN was selected because it preserves multivariate relationships among predictors and is suitable for mixed-type health data. Using multiple strategies enabled a sensitivity analysis to assess whether results were consistent across different assumptions about the structure of the missing data.

### 2.4. Chi-Squared Test and t-Test

To statistically assess the observed differences in asthma prevalence across demographic and socioeconomic subgroups, we conducted chi-squared tests for categorical variables and two-sample *t*-tests for continuous variables.

### 2.5. Logistic Regression

We conducted multivariable logistic regression analysis, suitable for modeling binary outcomes, to investigate factors associated with asthma among Hispanic participants. This allowed estimation of asthma odds based on demographic, socioeconomic, and health-related predictors, including age, sex, race, education, income, BMI, and access barriers. Categorical variables (sex, race, education, income, and access barriers) were coded using indicator functions against defined reference groups. The logistic regression quantified the independent contributions of each factor to asthma likelihood.

### 2.6. Multivariate Adaptive Regression Splines

We employed the MARS algorithm to capture complex nonlinear relationships and potential interactions among predictors. MARS is a flexible, non-parametric modeling technique that selects relevant variables and constructs piecewise linear basis functions, modeling nonlinear effects and interactions without manual transformations [19]. The MARS model utilized a binomial logistic link function for the binary asthma outcome. Predictor variables included age at survey, sex at birth, BMI, access barriers, education, and income, with education and income treated as ordinal numeric features based on their original categories. The method identifies optimal cut-points (“hinges”) for continuous variables, constructing additive and interaction terms reflecting localized effects. Specifically, the hinge function allows modeling changes in predictor-outcome relationships at specific thresholds.

In the MARS model, we fixed the random seed 121 to ensure reproducibility. We allowed interactions up to the two-way level and limited the model to a maximum of 100 candidate basis functions. A penalty value of 3 was used to discourage overly complex models, and a pruning threshold of 0.001 was applied to remove weak terms. The final model size was selected using 10-fold stratified cross-validation.

### 2.7. Conditional Inference Trees

We applied a CIT analysis to explore complex interactions and identify high-risk subgroups. This method is a non-parametric, recursive partitioning approach, selecting splitting variables based on statistically significant associations with the outcome, correcting for multiple testing [20]. The binary outcome was asthma status (yes/no), with predictors including BMI, age, sex, access barriers, education, and income. Categorical variables (sex, race, education, and access barriers) were included as factors, with education recoded numerically for ordinal analysis. The resulting tree provided interpretable splits based on significant variables, highlighting heterogeneity in asthma prevalence among subgroups. The resulting tree provided interpretable splits based on significant variables, highlighting heterogeneity in asthma prevalence among subgroups.

In the CIT model, we used settings that control splitting behavior and limit tree complexity. A quadratic test statistic was applied to evaluate potential splits. To reduce false-positive partitions, a Bonferroni-adjusted significance rule and a minimum criterion corresponding to a *p*-value of 0.05 were used. We also required at least 100 observations before a split could occur, and at least 50 observations in each terminal node, and we limited the tree depth to six levels.

All analyses were conducted in the *All of Us* Research Workbench JupyterHub environment using R version 4.5.0 (2025-04-11) running on a Linux (x86_64-pc-linux-gnu) platform.

## 3. Results

### 3.1. Sample Characteristics

There are 21,069 Hispanic participants in the study, including 2011 individuals with asthma and 19,058 without asthma, based on EHRs. Table 1 shows the asthma prevalence by key demographic/geographic subgroups among Hispanic participants. Additional results indicate significant associations between asthma status and several categorical predictors among Hispanic participants. Specifically, asthma prevalence differed significantly by sex (*p* < 0.001), race (*p* <0.001), education (*p* < 0.001), income (*p* < 0.001), access barriers (*p* < 0.001), and insurance type (*p* = 0.00103). In addition, the results in Table 2 showed that individuals with asthma were significantly older on average (mean age 49.6 vs. 45.2 years, *p* < 0.001) and had higher BMI (mean 32.3 vs. 28.9, *p* < 0.001). 

### 3.2. Missingness and Imputation

**Figure 1:**
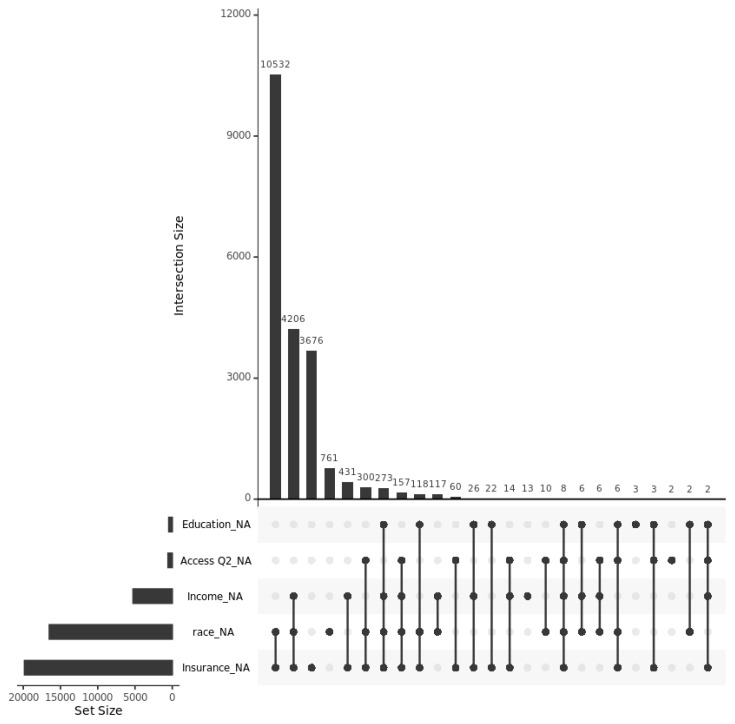
The missingness pattern of sociodemographic and healthcare access variables.

Missing values occur in some of the variables, as shown in Table 1. For two survey questions in Healthcare Access and Utilization Survey, there are around 2.71% and 2.31% missingness, respectively. The answer to another question is used to impute the other question, because they are related, and there are no missing values in access barriers. Insurance type is not in Table 1, and it has 19,852 missing values with 94.22% missingness. We included multiple ways to deal with missing values, including complete cases analysis, data creating an “Unknown” category, and using k-Nearest Neighbors (KNN) for the variables in Table 1. There are no missing values in age at survey and BMI among all these individuals. The missingness patterns of the variables are shown in Figure 1. Via different ways to deal with missing values, including complete cases, using ‘NA’ category for missing values and KNN imputation with k = 5, the core predictors such as age, sex, rural, BMI, education, and income are highly consistent across all three versions as shown in Table A1 and Table A2 (Appendix A). This stability indicates model robustness despite different handling of missing data.

### 3.3. Results for Statistical and Machine Learning Algorithms

The multivariate logistic regression findings, expressed as adjusted prevalence odds ratios (aPORs) with 95% confidence intervals (95% CIs) and *p*-values, are presented in Table 3, with all reference categories clearly indicated.

Age was significantly associated with asthma; each additional 10-year increase in age raised the odds of asthma by approximately 17.6% (aPOR = 1.176, 95% CI: 1.139–1.214, *p* < 0.001). Males had significantly lower odds of asthma compared with females (aPOR = 0.608, 95% CI: 0.543–0.681, *p* < 0.001), corresponding to a 39.2% reduction. Participants reporting access barriers had 26.3% higher odds of asthma than those without barriers (aPOR = 1.263, 95% CI: 1.114–1.433, *p* < 0.001).

Across income categories, a clear gradient was observed: higher income levels were associated with significantly lower asthma odds relative to the reference group. Adjusted odds ratios ranged from 0.82 for the 10–25k group (aPOR = 0.818, 95% CI: 0.696–0.964, *p* = 0.012) to 0.43 for those earning more than 150k (aPOR = 0.431, 95% CI: 0.353–0.528, *p* < 0.001), demonstrating a strong and consistent protective effect of increasing income. Each unit increase in BMI raised the odds of asthma by 2.9% (aPOR = 1.029, 95% CI: 1.023–1.035, *p* < 0.001).

Racial differences were partially significant. Compared with White participants (reference group), those classified as ‘Another Single Population’ did not show a statistically significant difference in asthma odds (aPOR = 1.08, 95% CI: 0.962–1.19, *p* = 0.184). In contrast, participants categorized as “More than One Population” had significantly higher odds of asthma, with an estimated 39.4% increase (aPOR = 1.39, 95% CI: 1.22–1.57, *p* < 0.001).

Education level showed a positive association with asthma diagnosis when compared to the reference group of participants with less than a high school education. Relative to this group, individuals with a high school diploma or GED had 77.0% higher odds (aPOR = 1.770, 95% CI: 1.495–2.095, *p* < 0.001), those with some college had 103.6% higher odds (aPOR = 2.036, 95% CI: 1.735–2.389, *p* < 0.001), and those with a college degree or higher had 94.8% higher odds (aPOR = 1.948, 95% CI: 1.651–2.300, *p* < 0.001). These results indicate that all three higher education groups differ from the “Less than High School” group. However, their corresponding confidence intervals overlap with one another, suggesting that the differences in asthma prevalence among the high school/GED, some college, and college graduate groups themselves (and not relative to the reference group) may not be statistically significant.

**Figure 2:**
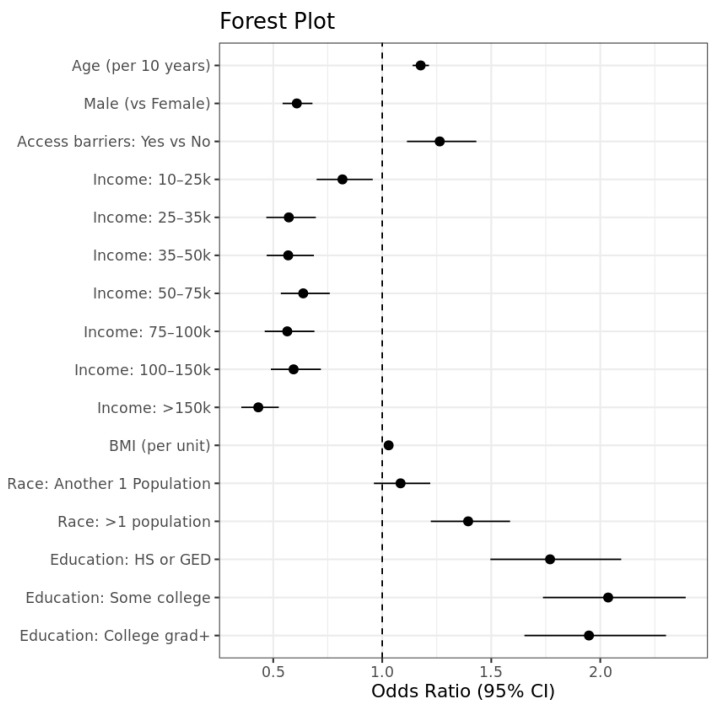
Forest plot showing adjusted odds ratios and 95% confidence intervals from the multivariable logistic regression model for asthma status.

The findings indicate that asthma status is significantly associated with age, sex, access barriers, BMI, income, race, and educational level, with higher income levels generally protective except for education, where increased diagnostic awareness may contribute to higher reported asthma prevalence (Figure 2).

Model diagnostics for logistic regression indicate satisfactory fit and no evidence of multicollinearity. The Hosmer–Lemeshow goodness-of-fit test yielded *X*^2^ = 12.13 with 8 degrees of freedom (*p* = 0.146), suggesting that the model’s predicted probabilities were consistent with the observed data. Variance Inflation Factor values ranged from 1.0 to 1.5 for individual predictors, all well below the conventional threshold of 5, indicating the absence of problematic multicollinearity (Appendix A Table A3). Overall, the logistic regression model demonstrates good calibration and reliable estimation of covariate effects.

**Figure 3:**
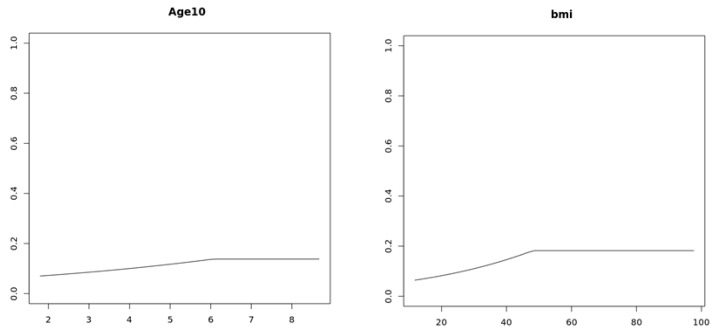
Partial dependence plots from the MARS model showing the marginal effects of Age (per 10 years) and BMI on asthma probability.

The MARS model was fitted using a logistic link function to model the binary asthma outcome, incorporating main effects and interactions up to second order. The model selected 14 basis functions (including the intercept) from the full set of candidate hinge and interaction terms, involving 7 of the 11 candidate predictors: BMI, age at survey, sex at birth, access, access barriers, education, and income. The fitted model is

$$\begin{array}{ll} log(\frac{\hat{p}}{1-\hat{p}})= & -1.066-0.523I_{\mathrm{Male}}-0.127\;h\left(6.06-\mathrm{Age}10\right)+0.304\;h\left(3-\mathrm{Income}\right)-0.104\;h\left(\mathrm{Income}-3\right)\\ & \;\;-0.0325\;h\left(48.1-\mathrm{BMI}\right)+6.697\;h\left(2.15-\mathrm{Age}10\right)I_{\mathrm{Male}}+0.239\;I_{\mathrm{Access}\;\mathrm{barriersYes}}\;h\left(3-\mathrm{Income}\right)\\ & \;\;-0.692\;h\left(2-\mathrm{Income}\right)I_{\mathrm{RaceWhite}}-0.0652\;h\left(\mathrm{Income}-2\right)I_{\mathrm{RaceWhite}}\\ & \;\;-0.461\;I_{\mathrm{Race}\;\mathrm{Another}\;\mathrm{Single}\;\mathrm{Population}}\;h\left(3-\mathrm{Education}\right)-0.298\;I_{\mathrm{RaceWhite}}\;h\left(\mathrm{Education}-3\right)\\ & \;\;-0.0492\;h\left(6.06-\mathrm{Age}10\right)h\left(5-\mathrm{Income}\right)+0.0526\;h\left(2-\mathrm{Income}\right)h\left(\mathrm{BMI}-48.1\right)\\ & \;\;+0.0687\;h\left(\mathrm{Income}-3\right)\;h\left(\mathrm{Education}-2\right)-0.974\;h\left(\mathrm{Income}-3\right)\;h\left(2-\mathrm{Education}\right), \end{array}$$

where h() is the hinge function and $I\left(\cdot \right)$ denotes the indicator function. The MARS logistic model revealed several nonlinear associations and interaction patterns related to asthma status. Males were associated with approximately 41% lower odds of asthma than females (aPOR ≈ 0.59). The relationship with age was nonlinear: individuals younger than about 60.6 years tended to have slightly higher modeled odds, with the association flattening at older ages. Income displayed a strong gradient, where participants in lower income brackets (<$35,000) exhibited 20–30% higher modeled odds, whereas higher income groups showed lower odds. Among individuals with BMI below 48.1, each unit increase in BMI corresponded to ≈ 3% higher estimated odds of asthma. A notable age–sex interaction indicated that younger males (≈<22 years) were modeled with substantially higher odds compared with older males. The access barrier–income interaction suggested that low-income individuals reporting more barriers to care had 25–30% higher modeled odds, highlighting socioeconomic heterogeneity. The race–education interaction indicated that individuals from smaller racial groups with lower education levels had 40–45% lower reported odds, which may reflect differences in diagnosis or reporting. Furthermore, combinations of low-income and high BMI were associated with about 5% higher modeled odds, while higher income and education levels coincided with a greater likelihood of asthma reporting. The marginal effects of age (10 years) and BMI estimated by the MARS model are given in Figure 3.

**Figure 4:**
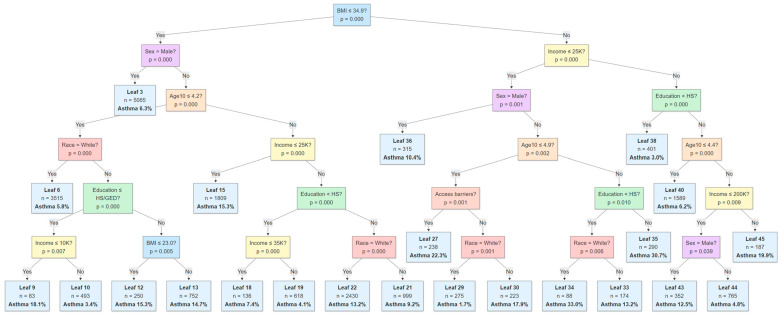
Classification results using the Conditional Inference Tree method to identify significant predictors of asthma among Hispanic individuals.

The CIT in Figure 4 identified BMI as the primary partitioning variable (*p* < 0.001), dividing participants with BMI ≤34.90 from those with higher values. Among individuals with lower BMI, sex further differentiated asthma status: males (Leaf 3, n = 5065) showed a low modeled asthma probability of ≈6%. For females, subsequent splits occurred by age (≤4.2 vs. >4.2 in Age10 units) and race, followed by education and income, revealing subgroups with asthma probabilities ranging from approximately 8% to 15%. Younger females who were non-White and had lower education generally showed moderate risk levels (≈9–12%), whereas older females with at least a high school education and lower income (Leaf 19, n = 136) reached higher probabilities around 14%. On the higher-BMI side (BMI > 34.90), the model next splits by income, followed by sex, age, and education, suggesting combined effects of socioeconomic and demographic characteristics. Females with higher BMI, lower education, and moderate-income levels exhibited asthma probabilities near 13%, while those with higher education but limited income (Leaf 40, n = 1589) had among the highest estimated probabilities at ≈20%. Conversely, males across BMI strata consistently displayed lower asthma probabilities (≈7–9%). Overall, the tree structure highlights complex interactions among BMI, income, education, race, and sex, with elevated asthma likelihood concentrated among older, obese females with at least a high school education but lower income, and the lowest rates among younger, lean males.

Both the MARS and conditional trees models identified consistent patterns in variable importance. Across both approaches, BMI, income, and education emerged as the most influential predictors of asthma status, reflecting the strong contribution of both biological and socioeconomic characteristics. The Random Forest model additionally highlighted age as an important factor, suggesting that age-related differences contribute meaningfully to the model’s predictive performance. Taken together, the results indicate that asthma status is primarily associated with a combination of biophysical (age, BMI) and socioeconomic (education, income) variables, while demographic attributes such as sex, race, and access barriers demonstrated comparatively smaller effects in explaining variability in asthma.

## 4. Discussion

This study investigated key factors associated with asthma prevalence in the Hispanic population using data from the *All of Us* Research Program. In particular, the study reveals interactions among factors that have not been examined in previous research on asthma in Hispanics, especially the interaction between access barriers, socioeconomic indicators such as education and income, and BMI.

Logistic regression analysis identified several significant predictors of asthma, including older age, female sex, increased access barriers, higher BMI, lower income, and higher education levels. Notably, Hispanic individuals living in areas with increased access barriers had 26.3% higher odds of having asthma, and each unit increase in BMI was associated with a 2.9% increase in asthma odds. Conversely, higher education levels were associated with higher odds of asthma. The finding contrasts with findings from the National Health and Nutrition Examination Survey, in which higher education levels among females were associated with lower asthma prevalence [21]. Therefore, the unusual association may be explained by factors such as enhanced knowledge and willingness to seek asthma care, more engagement with healthcare providers, as well as increased asthma diagnoses, compared to the group of participants with less than a high school education. Collectively, these findings support the presence of asthma disparities related to age, socioeconomic status, BMI, and access barriers within the Hispanic population [9,10,11,12,22,23].

The MARS model revealed nonlinear effects and interactions, such as increased asthma odds among younger individuals under 59.4 years and a slight decline in asthma with age for males over 21.9 years. The joint effect of low-income and high BMI also highlighted compounded vulnerability to asthma. The CIT identified BMI as the most influential predictor and further stratified asthma by age, sex, access barriers, education, and income. Elevated asthma prevalence was consistently observed among older, high BMI females with lower income and access barriers to care.

Such findings are consistent with age and sex differences in asthma prevalence. Throughout the life course, a shift in asthma occurs whereby females (9.6%) exhibit a greater likelihood of developing asthma in adulthood compared to males (6.3%) [22,23]. Additionally, the effects of access barriers, income, and BMI on increased asthma prevalence are supported by existing literature, underscoring the importance of addressing social determinants of health in medically underserved populations, including Hispanics [9,24,25,26].

A major strength of this study is the use of a large, diverse cohort from the *All of Us* Research Program, which provides more generalizable insights into asthma prevalence among Hispanic participants across varied socioeconomic backgrounds. By combining traditional statistical methods with interpretable machine learning techniques, such as MARS and CITs, we were able to capture complex, nonlinear relationships and interactions between risk factors, identifying nuanced risk profiles beyond what standard methods can reveal. Moreover, translating these complex relationships into meaningful determinant-outcome associations can inform public health interventions aimed at improving the health and well-being of Hispanic communities. Finally, incorporating social determinants of health, such as access barriers and socioeconomic indicators, provides a broad perspective on the challenges encountered by asthmatic Hispanics.

This study has several limitations. First, key environmental exposure measures, such as air pollutants and neighborhood allergens, are not yet comprehensively available in the *All of Us* database. Important variables, including mold exposure, precise geographic information, and lifestyle behaviors such as diet and physical activity, were also unavailable or highly incomplete. Because only the first three digits of participants’ zip codes are provided, we were unable to incorporate standard geographic or environmental classifications. This limitation may result in residual confounding, particularly in populations that experience higher exposure to environmental risks. Second, missing data and reliance on self-reported and EHR-derived information may introduce misclassification or bias in estimating asthma prevalence and related associations. Third, participation in *All of Us* is voluntary, which may introduce self-selection bias. Finally, all analyses are cross-sectional. Although our models identify associations between demographic, socioeconomic, and health-related factors and asthma, the study design does not allow for causal inference. Future studies should consider longitudinal approaches to clarify temporal and causal pathways that contribute to asthma disparities in minority populations.

## 5. Conclusions

Overall, among Hispanic participants in the *All of Us* Research Program, asthma prevalence is significantly influenced by age, sex, BMI, and access barriers. Individuals with higher BMI and those living in areas experiencing greater access barriers face higher odds of asthma, while males are less likely to have asthma than females. Older age is also associated with increased asthma prevalence. These results emphasize the importance of addressing structural and socioeconomic disparities in Hispanic communities. Public health interventions should prioritize equitable access to healthcare and consider integrated strategies targeting both asthma management and weight-related risk factors. These intervention strategies could include affordable transportation services, expanded clinics, and culturally appropriate physical activity programs to promote active living for Hispanic communities.

## Figures and Tables

**Table 1:** Asthma prevalence by key demographic/geographic subgroups among Hispanic participants (N = 21,069).

Variable	Subgroup	All N = 21,069 n (%)	Asthma (Yes) N = 2011 n (%)	Asthma (No) N = 19,058 n (%)
Sex	Female	14,772 (70.1%)	1573 (10.6%)	13,199 (89.4%)
	Male	6297 (29.9%)	438 (7.0%)	5859 (93.0%)
Access barriers	No	18,051 (85.7%)	1622 (9.0%)	16,429 (91.0%)
	Yes	3018 (14.3%)	389 (12.9%)	2629 (87.1%)
Race	Another Single Population *	957 (4.5%)	113 (11.8%)	844 (88.2%)
	More than One Population **	932 (4.4%)	132 (14.2%)	800 (85.8%)
	White	2660 (12.6%)	246 (9.2%)	2414 (90.8%)
	NA	16,520 (78.4%)	1520 (9.2%)	15,000 (90.8%)
Education Level	College Graduate/Advanced Degree	7320 (34.7%)	653 (8.9%)	6667 (91.1%)
	College: One to Three	5667 (26.9%)	620 (10.9%)	5047 (89.1%)
	Highest Grade: Twelve Or GED	3814 (18.1%)	390 (10.2%)	3424 (89.8%)
	Less than High School	3798 (18.0%)	291 (7.7%)	3507 (92.3%)
	NA	470 (2.2%)	57 (12.1%)	413 (87.9%)
Income	<$10,000	2413 (11.5%)	305 (12.6%)	2108 (87.4%)
	$10,000–$24,999	2916 (13.8%)	323 (11.1%)	2593 (88.9%)
	$25,000–$34,999	1827 (8.7%)	152 (8.3%)	1675 (91.7%)
	$35,000–$49,999	1995 (9.5%)	168 (8.4%)	1827 (91.6%)
	$50,000–$74,999	2236 (10.6%)	215 (9.6%)	2021 (90.4%)
	$75,000–$99,999	1453 (6.9%)	128 (8.8%)	1325 (91.2%)
	$100,000–$149,999	1525 (7.2%)	141 (9.2%)	1384 (90.8%)
	>$150,000	1438 (6.8%)	118 (8.2%)	1320 (91.8%)
	NA	5266 (25.0%)	461 (8.8%)	4805 (91.2%)

Note: n = number of participants in each subgroup; % = percentage of participants in each subgroup. * Another Single Population is defined as participants who identified as Asian, Black/African American, Middle Eastern or North African, Native Hawaiian or other Pacific Islander, or American Indian or Alaska Native. ** More than One Population included participants who identified as two or more races (i.e., White, Black, African American or African, Asian, Middle Eastern or North African, Native Hawaiian or other Pacific Islander, or American Indian or Alaska Native).

**Table 2:** Comparison of mean age and BMI between participants with and without asthma.

Variable	Asthma (Yes) Mean	Asthma (No) Mean	t-Statistic	*p*-Value
Age at Survey	49.6	45.2	−6.72	<0.001
BMI	32.3	28.9	−11.45	<0.001

**Table 3:** Multivariate logistic regression results for asthma status, presenting adjusted prevalence odds ratios (aPORs), 95% confidence intervals (95% CIs), and *p*-values with clearly defined reference categories.

Predictor	Level	Estimate	aPOR	95% CI	*p*-Value	Reference
Intercept	—	–4.018	—	—	<2 × 10^−16^	—
Age10	Per 10-year	0.162	1.18	1.14–1.22	<2 × 10^−16^	—
Sex	Male	–0.497	0.61	0.54–0.68	<2 × 10^−16^	Female
Access barriers	Yes	0.234	1.26	1.11–1.43	0.00026	No
Income	10 k–25 k	–0.202	0.82	0.70–0.96	0.0117	<10 k
	25 k–35 k	–0.560	0.57	0.47–0.70	2.96 × 10^−8^	<10 k
	35 k–50 k	–0.565	0.57	0.47–0.69	5.06 × 10^−9^	<10 k
	50 k–75 k	–0.450	0.64	0.53–0.77	4.65 × 10^−7^	<10 k
	75 k–100 k	–0.572	0.56	0.45–0.70	2.42 × 10^−8^	<10 k
	100 k–150 k	–0.522	0.59	0.48–0.73	1.04 × 10^−7^	<10 k
	>150 k	–0.840	0.43	0.35–0.52	<2 × 10^−16^	<10 k
BMI	per 1-unit	0.0288	1.03	1.02–1.04	<2 × 10^−16^	—
Race	Another Single Population	0.081	1.08	0.96–1.23	0.184	White
	More than One Population	0.332	1.39	1.22–1.59	5.66 × 10^−7^	White
Education	HS or GED	0.571	1.77	1.49–2.11	3.23 × 10^−11^	<High School
	Some College	0.711	2.04	1.74–2.39	<2 × 10^−16^	<High School
	College Grad or higher	0.667	1.95	1.65–2.31	3.14 × 10^−15^	<High School

## Data Availability

This study used data from version 8 of the *All of Us* Research Program’s Registered Tier Dataset, available to authorized users on the *All of Us* Researcher Workbench. All analyses and reporting were conducted in full compliance with *All of Us* Research Program data disclosure and privacy protection policies. Texas A&M University—Corpus Christi maintains an active Data Use and Registration Agreement (DURA) with the *All of Us* Research Program, which authorizes eligible researchers to access and use the *All of Us* Researcher Workbench for approved research activities.

## Appendix A

**Table A1:** Logistic regression estimates comparing different missing-data approaches: complete-case analysis, KNN imputation, and missing-indicator (“missing category”) modeling.

Variable	Complete Cases	KNN Imputed	Missing Category	Key Interpretation
(Intercept)	−3.356 ***	−3.992 ***	−3.807 ***	Baseline log-odds lowest after KNN.
Age10	0.131 ***	0.164 ***	0.164 ***	Age 10↑ odds ≈ 15%.
Male	−0.421 ***	−0.499 ***	−0.510 ***	Lower odds vs. female.
Access barriers (Yes)	0.317 *	0.248 ***	0.331 ***	Access/rural barrier ↑ risk ≈ 30%.
BMI	0.029 ***	0.029 ***	0.030 ***	Each BMI unit ↑ odds ≈ 3%.
Income 10–25 k	+0.038 ns	−0.178 *	−0.170 ·	Weak/negative association after imputation.
Income 25–35 k	−0.070 ns	−0.569 ***	−0.452 ***	Clear protective gradient.
Income 35–50 k	+0.026 ns	−0.557 ***	−0.431 ***	Higher income ↓ risk.
ncome 50–75 k	+0.033 ns	−0.418 ***	−0.291 **	Middle income ↓ risk.
Income 75–100 k	−0.426 ·	−0.605 ***	−0.408 ***	Declining risk continues.
Income 100–150 k	−0.003 ns	−0.458 ***	−0.322 **	Protective effect.
Income 150–200 k	−0.327 ns	−0.889 ***	−0.526 **	Strongest negative effect.
Income >200 k	−0.184 ns	−0.850 ***	−0.310 *	Consistent protective trend.
Income Unknown	—	—	−0.353 ***	Missing income ↓ risk.
Race (>1 pop.)	+0.215 ns	+0.266 **	+0.174 ns	Becomes significant after KNN.
Race White	−0.196 ns	+0.004 ns	−0.186 ns	No consistent pattern.
Race Unknown	—	—	−0.250 *	Slight negative association.
Education HS/GED	−0.063 ns	+0.558 ***	+0.493 ***	Higher education ↑ reported asthma.
Education Some College	+0.021 ns	+0.694 ***	+0.579 ***	Positive association across models.
Education College Grad +	−0.091 ns	+0.644 ***	+0.479 ***	Awareness/diagnosis effect.
Education Unknown	—	—	+0.672 ***	Missing education ↑ asthma odds.

Note: Significance: · *p* < 0.10; * *p* < 0.05; ** *p* < 0.01; *** *p* < 0.001. ns: not significant. The symbols ↑ and ↓ denote an increase and a decrease, respectively. Race (>1 pop.) indicates participants belonging to more than one racial population.

**Table A2:** Comparison of logistic regression results across three missing data approaches: complete-case analysis, KNN imputation, and missing-indicator (missing category) modeling.

Aspect	Complete-case	KNN (Imputed)	Missing Category	Interpretation
Sample size (N)	4031	21,047	21,047	KNN/Unknown retain full data; base model loses ≈80% due to missingness.
Age10	+0.131 ***	+0.164 ***	+0.164 ***	Very stable; every 10 yr ↑ asthma odds ≈ 14–18%.
Male	−0.421 ***	−0.499 ***	−0.510 ***	Males have ≈40–45% lower odds.
Access barriers (Yes)	+0.317 *	+0.248 ***	+0.331 ***	Rural residence/access barriers ↑ risk 25–40%.
BMI	+0.029 ***	+0.029 ***	+0.030 ***	Each BMI unit ↑ odds ≈3%.
Income pattern	Weak, non-significant	Strong negative gradient (*p* < 0.001)	Negative gradient + Unknown category significant	Imputation clarifies consistent socioeconomic gradient.
Education	NS	Strongly positive (College > HS > below HS)	Strongly positive; “Unknown” also ↑ risk	Higher education linked to diagnosis/awareness; missing education also predictive.
Race effects	Not significant	“More than one population” ↑ risk (*p* < 0.001)	“Unknown race” ↓ risk (*p* < 0.05)	Imputation enhances racial contrasts; small subgroups affect base estimates.
Precision (SEs)	Large	Small	Small	Imputation stabilizes SEs; tighter confidence intervals.

Note: * *p* < 0.05; *** *p* < 0.001. The symbols ↑ and ↓ denote an increase and a decrease, respectively. SEs refers to the degree of standard errors.

**Table A3:** Variance inflation factors (VIFs) and degrees of freedom (df) for predictors in the multivariate logistic regression model.

Metric	Age10	Sex	Access Barriers	Income	BMI	Race	Education
VIF	1.082	1.023	1.094	1.362	1.032	1.201	1.412
df	1	1	1	8	1	2	3
